# Notes and News

**Published:** 1888-01-21

**Authors:** 


### NOTES AND NEWS.

COLLECTED AND COMMUNICATED.

LORD CHARLES BERESFORD has fixed Wednesday, the 22nd of February, at 3 o'clock, for the annual Court of Governors of the Seamen's Hospital Society. The Court will be held at the Whitehall Rooms, Hotel Métropole.

THE French Government has, through its ambassador, M. Waddington, sent a donation of £2,000 to the building fund of the French Hospital. M. Waddington has added a personal donation of £100.

UP to the present time 170 applicants have applied for the appointment of secretary of the London Hospital, in the place of Mr. A. H. Haggard, resigned. Of these the committee have selected eight gentlemen, and the election of one of them will take place on Monday next, 23rd inst.

THE Right Hon. Lord Brassey will preside at the 14th annual meeting of the Hospital Saturday Fund, to be held at the Memorial Hall on February 8th next. The following are also expected to attend :—Col. Sir E. Y. W. Henderson, K.C.B., Lord Francis Hervey, Mr. G. C. T. Bartley, M.P., Sir E. Hay Currie, Sir Morell Mackenzie, and Col. Chas. Mercier.

A MEETING of the Council of The Hospitals Association was held at Norfolk House on Tuesday, the 17th inst., Dr. Bristowe, F.R.S., in the chair. Among those present were : Mr. F. C. Carr-Gomm (Chairman of Committee of the London Hospital), Mr. T. Holmes, F.R.C.S., Major-General Keating, V.C., K.C.B., Dr. J. C. Steele (Guy's Hospital), Dr. Gilbart Smith, F.R.C.P., Mr. A. H. Haggard, Lieut.-Colonel Montefiore, Mr. Henry C. Burdett, Mr. Keith Young, F.R.I.B.A , Dr. G. W. Potter, Mr. P. Michelli, and others. The meeting expressed by resolution its cordial welcome to Dr. Bristowe on his acceptance of the presidency of the Association, and its high appreciation of the honour conferred upon the Council and members by his appointment. Dr. Bristowe disclaimed any special merit in yielding to the request of the Council, and stated that he had felt impelled by a sense of duty to accept the honour offered to him. He considered that the Association had done much valuable work for hospitals and all connected with them, and specially instanced the National Pension Fund, and the Nurses' Registration Scheme now approaching completion. He also emphasised his conviction that the Association was steadily growing in the esteem and confidence of the hospital world and of the public, and that the recent additions to the council of Sir E. Hay Currie, Mr. Carr-Gomm (of the London Hospital), and Mr. E. H. Lushington (treasurer of Guy's) would add materially to its strength and judicial authority. Members of the C ouncil expressed their especial gratification that through the admirable energy and perseverance of Mr. Henry C. Burdett the £20,000 needed to establish the National Pension Fund on a firm financial basis had finally been secured.

HOPES are now entertained that the epidemic of small pox at Leeds may be stamped out, and Chief Superintendent Newhouse and his staff are unceasing in their quest for new cases. As a matter of precaution, however, the sanitary authorities have begun to build a new temporary hospital of wood, the original small-pox hospital at Burmantofts being nearly full.

THE Victoria Infirmary, Glasgow, recently lost one of its most earnest supporters by the death of Mr. Robert Couper, of Milnhome. That gentleman has, indeed, left the residue of his estate to the infirmary, but in meanwhile it is enjoyed in life-rent by his widow. Mrs. Couper, however, who shared her husband's interest in this good work, has generously offered to give up at once £10,000 of the money thus left to her. This handsome gift will permit the building of the new hospital to be commencd forthwith.

THE Maharajah of Darbhanga, in Bengal, has established a hospital and dispensary for female patients near his ancestral seat in the district of Darbhanga, Behar, and is erecting new quarters for its accommodation at a cost of Rs. 55,000, in connexion with Lady Dufferin's Medical Aid for Women Fund. This announcement reached England by the last mail; and to those who can remember India as it was some twenty or thirty years ago, this novel form of benefaction will serve as a striking reminder of the great social changes that have taken place in that country during the period that India has been "the India of the Queen." It is also one of many illustrations that recent mails have brought home of the firm hold that the movement for medical aid to the women of India, inaugurated by the Countess of Dufferin, and specially encouraged by her Majesty the Queen, has taken on the imagination and sympathy of the Princes and people of India. Probably few persons in this country, however, will be aware that this liberal and enlightened gift is only the latest, and one of the least considerable, of a long series of philanthropic acts on the part of this great Hindoo noble, which have been on a scale of magnificence hardly ever equalled.

NOT many diseases are more painful than rheumatism; none, perhaps, is at once so trying and so common. Of all places in England Buxton is the most frequented by those who suffer from this complaint, its baths having a long-established and well-grounded fame; and by far the larger number of cases treated at the Devonshire Hospital and Buxton Bath Charity during last year have been sufferers from one form or another of rheumatism or gout. Of the 2,389 cases under treatment during the year 1887, over 2,000 have come under this head, in 312 cases complicated with heart disease. This charity was founded in 1858 by the Duke of Devonshire, and its usefulness was greatly increased by the grant of £24,000 from the Cotton Districts Convalescent Fund, the governors of which have a prior claim in recommending patients to 150 beds. Subscriptions to a considerable amount are usually received through the numerous hotels and lodging-houses in Buxton, visitors who have received benefit at the Derbyshire Spa desiring thus to help sufferers less rich in worldly goods than themselves; but this source of income has been diminishing of late years in proportion to the prevailing pecuniary depression, and the usefulness of the charity, once wholly and still largely dependant on these contributions, has thus been impaired. The institution has, indeed, many friends who expend both time and money in making the necessarily prolonged stay of the inmates pleasant as well as beneficial; and the great majority of them, even when the disease is of a nature that cannot be cured, depart relieved and grateful.

THE following vacancies are announced this week, the amount of salary in each case being given after the name of the institution :—

**Resident Medical Officers.**

Birkenhead Borough Hospital. Senior House Surgeon.—£80, without extras.
Bristol Hospital for Sick Children and Women. House Surgeon. —£100, with rooms and attendance.
Bristol General Hospital. Physician's Assistant.—£50.
Huddersfield Infirmary. Junior House Surgeon.—£40.
Radcliff Infirmary, Oxford. House Surgeon —£80.
Birmingham General Hospital. Two Assistant House Surgeons. —Board, etc., no salary.
Brixton, Streatham, and Herne Hill Dispensary. Resident House Surgeon.—£100, with rooms, attendance, coal, and gas.
Macclesfield General Infirmary. Junior House Surgeon.—£70.

**Non-resident Medical Officers.**

Royal Hospital for Diseases of the Chest, City Road, E.C. Physician.
Bromley Union, Kent District. Medical Officer.—£60, with vaccination fees and extras, about £15.
Hospital for Sick Children, Great Ormond Street, W.C. Medical Registrar and Pathologist.—An honorarium of 50 guineas at end of each year. Clinical Assistant in the Out-patient Department.

**Matrons.**

Teignmouth, Dawlish, and Newton Infirmary and Convalescent Home. Matron, thoroughly trained both as a Surgical and Medical Nurse; also able to superintend the housekeeping. —£35.
Fisherton House Asylum. Matron, who has had some hospital training preferred; musical, and about 30 years of age.

**Nurses.**

Samaritan Free Hospital. Surgical Nurse, age 22 to 35; preference given to a total abstainer.
Buckinghamshire General Infirmary. Charge Nurse for women's ward; must understand both Medical and Surgical work; age not over 35.—£25 a year, with indoor uniform
Teignmouth Infirmary. Thoroughly trained Medical and Surgical Nurse.—State salary expected.
St. Saviour's Union Infirmary. Ward Nurses, hospital experience essential.—£21, rising by £1 annually to £25, with uniform.
Havant Union. Nurse and Needlewoman. Age from 35 to 45 years.—£25, with rations and uniform.
Workhouse Infirmary Nursing Association, 6, Adam Street, Adelphi. Trained Nurse, under 40. Apply to Lady Superintendent.
Kingston Nurses' Home Hull. Trained Monthly Nurse.
Cancer Hospital, Myrtle Street, Liverpool. Trained Nurse. Apply to Matron.

**Probationers.**

The Hospital, Saffron Walden. Probationer, not under 20.— Salary after first three months, £10 a year.
Worthing Infirmary. Probationer wanted.—No salary first year, all found.

---

IN accordance with annual custom, a special children's service was held in Christ Church, Waterloo, Liverpool, on Sunday, the 8th inst. (Hospital Sunday), when after the singing of a special Litany, gifts were received for the sick children in the various hospitals and orphanages in the city. Nearly four hundred gifts were received, including toys, pictures, scrap-books, flowers, and fruit. After the presentation, which took about half-an-hour, the Rev. T. K. Dickson gave a short address on the claims of hospitals on children, illustrated by some true stories of child-life in hospital, and the interesting service was brought to a conclusion with an appropriate hymn and the benediction.

---

THE Royal Cornwall Infirmary has been for a year without a physician. A difference of opinion then took place between the visiting physician and the house surgeon regarding their respective duties, in consequence of which the physician resigned. Since that time the institution has been going on as well as it can without that official. Whether that is well or ill it is not for us to say; but as indicating the current of public opinion we may mention that it has been decided not to hold the usual Hospital Sunday collections in several places of worship, because of this irregularity. According to the rules of the infirmary, it should never be without a physician, and the failure of the management to comply with the laws of their institution is evidently shaking public confidence in their wisdom

THERE are about fifteen labour colonies (arbeiten colonien), similar in principle to the "home colonisation" scheme now talked of, scattered over Germany. The earliest of these was that of Wilhelmsdorf in Westphalia, instituted in 1882 by Herr von Bodelschwingh, where 350 persons are now occupied in the cultivation of 800 acres of land. Similar institutions exist in Steenwyk in East Holland, where 5,000 acres of land are occupied by 1,800 persons, under regular conditions of gradual repayment for the benefits and advantages offered. The movement is steadily extending, and is everywhere eminently successful. It can hardly be doubted that a similar principle applied in this country would tend greatly to the well-being and prosperity of many, both in town and country, who are now on the verge of starvation.

---

THE *Newcastle Chronicle* says: "The death occurred on the 12th inst., at the Royal Infirmary, Newcastle, of Nurse McIntyre, better known as Nurse Ellen. She had been engaged as nurse for nearly a quarter of a century, principally in the Magdalen Ward of the infirmary, and was not only distinguished for her faithfulness as a nurse during all that time, but for the consideration she manifested in the welfare of the young women after they had passed from under her special care. Yesterday a service was held in the ward, conducted by the Rev. J. Mitchell, chaplain of the infirmary, at which all the nurses who could be spared from duty were present. Mr. J. Holland, city missionary, who had been accustomed to visit the patients under treatment in the ward for a period of nearly twenty-one years, referred to the long services of the departed nurse, and to her devotion to the well-being of those who had been placed under her care. After the service, the remains were removed to the Central Station, to be conveyed to Leith for interment there. A beautiful wreath, given by the nurses as a last tribute of affection, was laid upon the coffin. Nurse Ellen was seventy-four years of age."

---

THE important work done by hospitals themselves should not make us forget the complementary work undertaken by convalescent homes. It is impossible, with the demands continually made on hospital resources, to keep patients till they have wholly recovered strength as well as health; they must go to make room for more urgent cases, and often the premature return to a home whose comforts are few at the best of times, and are perhaps lessened by the illness of the breadwinner, causes the return of the lost vigour to be long delayed, or, it may be, never regained; whereas a week or two spent at a convalescent home in pure and bracing air would at once complete recovery. These convalescent establishments are as yet a modern branch of our great and ancient hospital system, and the good they do is therefore less known and appreciated than it should be. One of the most deserving of these is the "Morley House" seaside home for convalescent London workmen. Established in 1882 at the suggestion of the late Mr. Samuel Morley, this institution is largely supported by the working-men of London, who also, by means of delegates from workshops and friendly societies, have a considerable share in its management. It has indeed had generous friends. Besides Mr. Morley, grateful mention should be made of the late Lord Wolverton, Postmaster-General, who gave £1,000 to the home with a view to enabling the hard-worked postmen to share in its benefits when invalided. Another generous friend is the treasurer (Mr. H. N. Hamilton Hoare), through whose aid the committee were enabled to purchase their present home and grounds at St. Margaret's, near Dover. In spite of all this, however, the institution still owes £800 to its bankers, and with increased means could do more good; and the creditable efforts made by working men to support it, make it not less but more deserving of aid from the charitable public.

